# Plant Growth-Promoting Rhizobacteria as Tools to Improve the Growth of Kohlrabi (*Brassica oleracea* var. *gongylodes*) Plants in an Aquaponics System

**DOI:** 10.3390/plants13050595

**Published:** 2024-02-22

**Authors:** María Carmen Piñero, Jacinta Collado-González, Ginés Otálora, Josefa López-Marín, Francisco M. del Amor

**Affiliations:** Department of Crop Production and Agri-Technology, Instituto Murciano de Investigación y Desarrollo Agrario y Medioambiental (IMIDA), C/Mayor s/n, 30150 Murcia, Spain; jacinta.collado@carm.es (J.C.-G.); gines.oralora@carm.es (G.O.); josefa.lopez38@carm.es (J.L.-M.)

**Keywords:** red tilapia, purple kohlrabi, nitrates, organic fertilization, aquaponics system, circular economy framework

## Abstract

The use of nitrogen as a fertilizer can be highly risky when used excessively, and it is therefore necessary to find novel techniques to reduce its use. Aquaponics reduces the use of synthetic fertilizers and water, and the leaching of nitrate into the environment. One way to avoid problems due to a reduction in nitrogen availability could be the use of plant growth promoting rhizobacteria (PGPR). This study examines the effect of PGPR on kohlrabi plants grown with a traditional nutrient solution (100S), in combination with “fish water” (50F/50D), or with a supplement of synthetic fertilizers (50F/50D + S). Two formulations were used: T1 (*Azospirillum brasilense* and *Pantoea dispersa*) and T2 (*Azotobacter salinestris*). Irrigation with 50F/50D caused a reduction in several of the measured parameters. The combined application of 50F/50D with T1 attenuated the negative effects. T2 did not present significant effects on the parameters measured. The results obtained with 50F/50D + S hardly showed differences with the 100S. Thus, by irrigating with 50F/50D + S, we were able to maintain the yields while reducing fertilizer use and water. The combined use of T1 and 50F/50D was also positive; however, it would be necessary to continue adjusting the amount of nitrate supplied to maintain production.

## 1. Introduction

Nitrogen (N) is an important component of synthetic fertilizers, as it is one of the most utilized nutrients by plants, but it is also one of the most expensive inputs in economic and environmental terms [1]. Nevertheless, farmers usually provide it in large quantities, sometimes in excess, to maximize crop yields, which carries many risks at the levels of the environment and human health [1]. At an environmental level, the most frequent impact observed due to the misuse of nitrogenous fertilizers is the contamination of surface and underground water resources due to the leaching of nitrates (NO_3_^−^), which causes the eutrophication of freshwater and marine ecosystems [2]. In addition, they can generate toxic emissions of ammonia (NH_3_) when gaseous N oxides react with the ozone in the troposphere [3,4]. On the other hand, at the level of human health, the danger lies in the fact that once NO_3_^−^ is ingested, it is quickly transformed into nitrites (NO_2_^−^) and N-nitroso compounds. These compounds may be responsible for diseases such as methemoglobinemia, which is dangerous for children, and cancer, due to the transformation of NO_2_^−^ into amines and amides inside the human body [2]. Therefore, it is necessary to find cultivation methods to correctly manage N fertilization.

Aquaponics could be a good option, as this technique reduces the use of synthetic fertilizers and water, as aquaculture products are re-utilized within a circular economy framework, thereby guaranteeing its low environmental impact and good sustainability [4]. Production through aquaponics systems would not only reduce the use of synthetic fertilizers, but it could also completely eliminate NO_3_^−^ waste into the environment [5]. The most advanced aquaponics systems have a biological filter, where the NH_3_ excreted by the fish is converted into NO_2_^−^ and then into NO_3_^−^ [6]; however, one of the problems of this type of system is that some mineral nutrients (such as iron, calcium, potassium, phosphorus, or manganese) may be deficient for the correct growth of the crop, and some NO_3_^−^ deficiencies have also been observed [6].

N is involved in many aspects of plant development, such as growth, photosynthesis, stomatal conductance, the maximum potential quantum efficiency of photosystem II, and chlorophyll content [7]. Therefore, a reduction in the availability of N for plants could result in a reduction in their growth and yield [8]. One way to solve this problem could be through the use of plant growth promoting rhizobacteria (PGPR), which fix atmospheric N and improve the absorption of water and minerals [4]. Ahemad and Kibret [9], listed some the functional activities of PGPR, among which we find (i) the ability to increase the availability of nutrients to plants, (ii) the promotion of plant growth, generally through phytohormones, (iii) the ability to degrade organic pollutants, and (iv) the control of diseases, through the production of antibiotics and antifungal metabolites. Thus, PGPR could be a good alternative to traditional mineral nutrition, as they contribute to plant growth and contamination avoidance.

The role of *Azotobacter* and *Azospirillum* as PGPR is due to two main mechanisms, nitrogen fixation and phytohormone production. Some of these hormones cause changes in the roots, thus improving the absorption of minerals and inducing higher yields, even with a lower contribution of synthetic fertilizers [10,11]; however, authors such as Muthukumar and Udaiyan [12] observed that co-inoculation of *Pantoea dispersa* with *Azospirillum* was more successful.

Given the above, the use of sustainable and environmentally friendly cultivation techniques, such as aquaponics and PGPR systems, is of great relevance. The combination of these two systems will not only have beneficial effects on the environment, but the reduction in the use of synthetic fertilizers and water will also result in economic benefits for the farmer.

To do this, this study will be carried out on kohlrabi plants, which belong to the *Brassicaceae* family. It is a vegetable with great nutritional value due to its high content of antioxidants such as vitamin C, glucosinolates, phenolic compounds and nutrients such as potassium, which have anti-inflammatory, antioxidant and anti-cancer properties [13].

To the best of our knowledge, this is the first study on the use of PGPR in kohlrabi plants grown under an aquaponics system. Thus, the main goal of this work was to study the effect of the application of two different commercial formulations with PGPR to alleviate the stress caused by the low doses of N provided in aquaponics systems. For this, their effect on weight, anion concentration, gas exchange, content of chlorophylls, and sugar content, lipid peroxidation, and total phenols in kohlrabi leaves was analyzed. The results from this study could help produce kohlrabi plants using innovative aquaponics systems combined with the use of PGPR, to greatly reduce the environmental impacts of intensive horticulture.

## 2. Results

### 2.1. Plant Material and Growth Conditions

**Figure 1:**
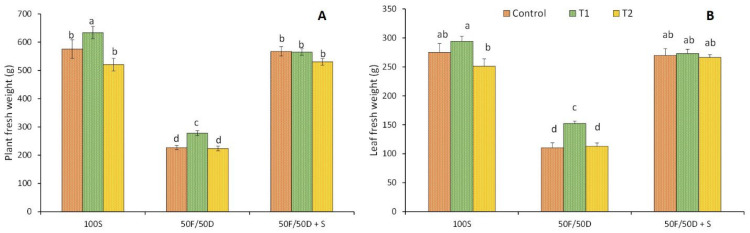
Plant fresh weight (**A**) and leaves (**B**) of kohlrabi plants irrigated with control solution (100S), 50% fish water + 50% drainage water (50F/50D) and 50% fish water + 50% drainage water + synthetic fertilizer (50F/50D + S); and inoculated with two formulations of plant growth-promoting rhizobacteria (Control, T1 and T2). Data are presented as treatment means ± SE (*n* = 6). Different letters indicate significant differences (*p* ≤ 0.05) between treatments.

At the end of the experiment, the kohlrabi plants grown with the fish water and drainage water (50F/50D) mix were reduced in size, 247.2 g FW, with respect to control plants, which obtained a mean weight of 575.5 g FW (100S) (Figure 1A). The same result was observed in the fresh weight of the leaves, which decreased from 275.2 g to 110.3 g FW (Figure 1B); however, the 50F/50D + S treatment did not significantly affect either the plant weight or leaf weight (Figure 1A,B). On the other hand, the plants inoculated with T1 increased in weight both in combination with 100S, and with the 50F/50D treatment (Figure 1A,B). In the case of the 100S treatment, the increase was 9.2% in the plant fresh weight, and 6.5% in the leaf fresh weight, and with the 50F/50D treatment, the increase was 18.4% in the plant fresh weight, and 27.6% in the leaf fresh weight, as compared to the plants irrigated with the same treatment but without PGPR (Figure 1A,B).

### 2.2. Ion Determination

**Figure 2:**
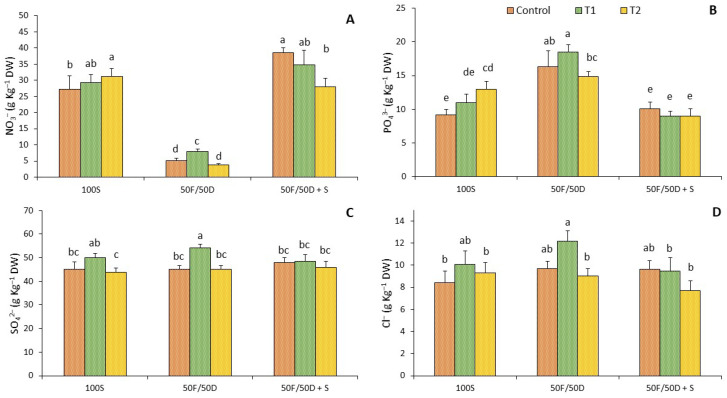
Anion concentrations: nitrate (**A**), phosphate (**B**), sulfate (**C**) and chlorides (**D**) in kohlrabi plants irrigated with control solution (100S), 50% fish water + 50% drainage water (50F/50D) and 50% fish water + 50% drainage water + synthetic fertilizer (50F/50D + S); and inoculated with two formulations of plant growth-promoting rhizobacteria (Control, T1 and T2). Data are presented as treatment means ± SE (*n* = 6). Different letters indicate significant differences (*p* ≤ 0.05) between treatments.

The NO_3_^−^ concentration in the tissues was drastically lower in the plants irrigated with the 50F/50D treatment, from 27.2 in the control, to 5.2 g Kg^−1^ DW (Figure 2A). On the contrary, in the plants irrigated with the 50F/50D + S treatment, the values were significantly higher than in the control plants (38.5 g Kg^−1^ DW) (Figure 2A). The PGPR had different effects depending on the irrigation treatment with which they were combined. In the case of T1, it only caused a significant increase when combined with the 50F/50D treatment (Figure 2A). And in T2, the NO_3_^−^ concentration increased in combination with the 100S, but it was reduced in combination with the 50F/50D + S treatment (Figure 2A). The concentration of phosphates (PO_4_^3−^) increased both in the 50F/50D irrigation treatment, and in T2 treatment in combination with the 100S treatment (Figure 2B). The rest of the treatments did not have significant effects on the PO_4_^3−^ values in the leaves. On the other hand, the concentration of sulfates (SO_4_^2−^) showed a tendency to increase when the plants were inoculated with T1, but significant differences were only observed when combined with the 50F/50D treatment (Figure 2C). In the case of Cl^−^ concentration, no significant effect was observed in any of the treatments applied (Figure 2D).

### 2.3. Gas Exchange 

**Figure 3:**
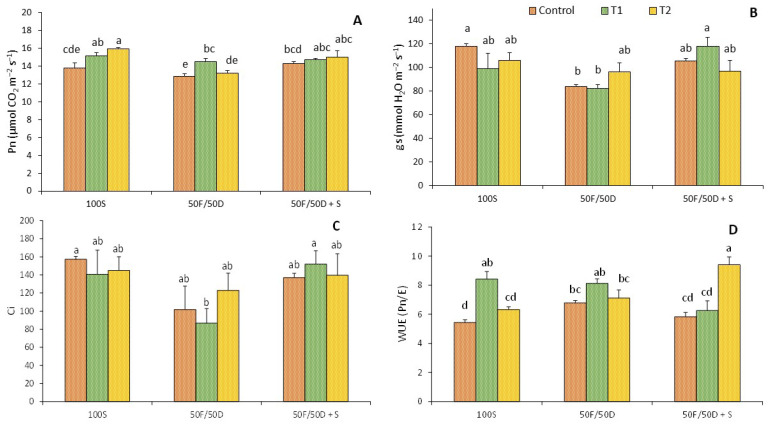
Net CO_2_ assimilation rate (Pn) (**A**), stomatal conductance (gs) (**B**), internal CO_2_ concentration (Ci) (**C**) and WUEi (Pn/E) (**D**) in kohlrabi plants irrigated with control solution (100S), 50% fish water + 50% drainage water (50F/50D) and 50% fish water + 50% drainage water + synthetic fertilizer (50F/50D + S); and inoculated with two formulations of plant growth-promoting rhizobacteria (Control, T1 and T2). Data are presented as treatment means ± SE (*n* = 6). Different letters indicate significant differences (*p* ≤ 0.05) between treatments.

The Pn increased in the plants inoculated with PGPR. In the case of plants irrigated with the 100S treatment, Pn increased from 13.8 µmol CO_2_ m^−2^ s^−1^ to 15.1 µmol CO_2_ m^−2^ s^−1^ in the case of T1, and to 15.9 µmol CO_2_ m^−2^ s^−1^ in the case of T2 (Figure 3A). In the plants irrigated with the 50F/50D treatment, the increase was from 12.9 µmol CO_2_ m^−2^ s^−1^ to 14.5 µmol CO_2_ m^−2^ s^−1^ when T1 was added. The rest of the combinations did not have significant effects on Pn (Figure 3A). The gs was reduced by 28.9% in the plants grown with the 50F/50D treatment, as compared to the control plants. However, when this irrigation treatment was combined with T2, this reduction disappeared, and the values of the control plants were maintained (Figure 3B). In Figure 3C, it can be observed that Ci showed a similar behavior to gs in all the treatments. The application of the 50F/50D treatment increased the WUE of the plants with respect to the control plants, from 5.4 to 6.8 (Figure 3D). On the other hand, the application of T1 increased the WUE in plants irrigated with the 100S treatment, from 6.8 to 8.1, and T2 increased it in plants irrigated with the 50F/50D + S treatment, from 5.8 to 9.4 (Figure 3D).

### 2.4. Chlorophylls

**Figure 4:**
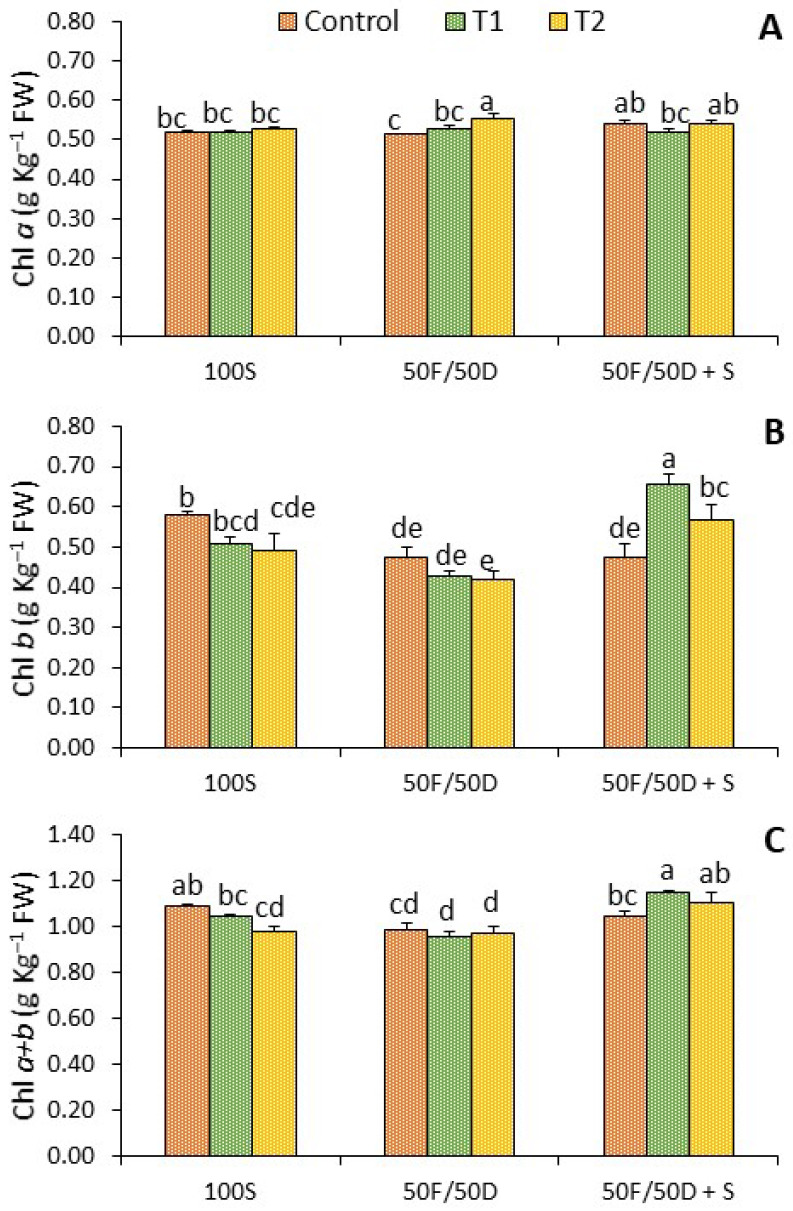
Chlorophyll *a*; (**A**) chlorophyll *b*; (**B**) chlorophyll *a* + *b* (**C**) in kohlrabi plants irrigated with control solution (100S), 50% fish water + 50% drainage water (50F/50D) and 50% fish water + 50% drainage water + synthetic fertilizer (50F/50D + S); and inoculated with two formulations of plant growth-promoting rhizobacteria (Control, T1 and T2). Data are presented as treatment means ± SE (*n* = 6). Different letters indicate significant differences (*p* ≤ 0.05) between treatments.

Chlorophyll *a* levels were increased from 0.51 g Kg^−1^ FW to 0.55 g Kg^−1^ FW in plants irrigated with the 50F/50D treatment with the application of T2, as compared to those irrigated with this same nutrient solution without PGPR (Figure 4A). No differences were found between the other treatments. Chlorophyll *b* levels were reduced by the 50F/50D and 50F/50D + S treatments, from 0.58 g Kg^−1^ FW to 0.48 g Kg^−1^ FW, in both cases, and by the application of T2 combined with the 100S treatment (from 0.58 g Kg^−1^ FW to 0.49 g Kg^−1^ FW) (Figure 4B). On the contrary, T1 combined with the 50F/50D + S treatment caused an increase of 27.5% as compared to plants irrigated with the same treatment but without PGPR (Figure 4B). However, the sum of chlorophyll *a* and *b* (*a* + *b*) was reduced only in plants irrigated with the 50F/50D treatment (9.5%), and those treated with 100S in combination with T2 (10.2%) (Figure 4C). In addition, this value increased by the combination of T2 and treatment 50F/50D + S (8.8%), reaching the same values as the control plants (plants irrigated with the 100S treatment and without PGPR) (Figure 4C).

### 2.5. Total Soluble Sugars

**Figure 5:**
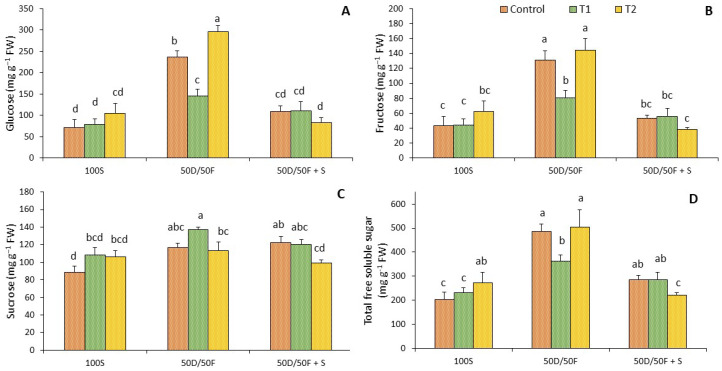
Glucose (**A**), fructose (**B**), sucrose (**C**) and total free soluble sugars (**D**) in kohlrabi plants irrigated with control solution (100S), 50% fish water + 50% drainage water (50F/50D) and 50% fish water + 50% drainage water + synthetic fertilizer (50F/50D + S); and inoculated with two formulations of plant growth-promoting rhizobacteria (Control, T1 and T2). Data are presented as treatment means ± SE (*n* = 6). Different letters indicate significant differences (*p* ≤ 0.05) between treatments.

The total sugar content varied from 202.8 mg g^−1^ DW to 504.2 mg g^−1^ DW (Figure 5D). The sugar content in kohlrabi plants in the different treatments is shown in Figure 5. The application of the 50F/50D treatment increased the contents of glucose (70.0%), fructose (67.1%), sucrose (24.1%), and total free soluble sugars (58.2%), as compared to the control plants (Figure 5A–D). However, the 50F/50D + S treatment only increased the values of sucrose (27.7%) and total free soluble sugars (28.7%) (Figure 5C,D). The application of T1 had significant effects only when it was applied in combination with the 50F/50D treatment, causing a reduction in both glucose and fructose levels (38.6% and 38.7%, respectively), and therefore, in total free soluble sugars (25.0%) (Figure 5A,B,D). On the contrary, T2 combined with the 50F/50D treatment increased glucose (50.8%); and when combined with 50F/50D + S, it reduced sucrose levels and total free soluble sugars (22.0% and 18.7%, respectively) (Figure 5A,C,D).

### 2.6. Lipid Peroxidation and Total Phenolic Compounds

**Figure 6:**
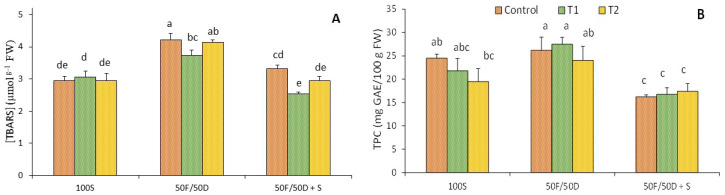
Lipid peroxidation (TBARS) (**A**) and total phenol compounds (TPC) (**B**) in kohlrabi plants irrigated with control solution (100S), 50% fish water + 50% drainage water (50F/50D) and 50% fish water + 50% drainage water + synthetic fertilizer (50F/50D + S); and inoculated with two formulations of plant growth-promoting rhizobacteria (Control, T1 and T2). Data are presented as treatment means ± SE (*n* = 6). Different letters indicate significant differences (*p* ≤ 0.05) between treatments.

The amount of TBARS detected in the plants grown with the 50F/50D treatment (4.21 µmol g^−1^ FW) was significantly higher than that detected in the plants grown with the 100S treatment (2.94 µmol g^−1^ FW) (Figure 6A). Inoculation with T1 caused a reduction in TBARS in plants irrigated with the 50F/50D treatment (11.4%) and the 50F/50D + S treatment (23.5%) (Figure 6A). Kohlrabi leaves showed values of total phenolic compounds (TPC) that ranged between 16.2 and 17.5 mg GAE 100 g^−1^ FW (Figure 6B). The only significant differences in this parameter were observed in the plants grown with the 50F/50D + S treatment, which showed lower values than the control plants.

## 3. Discussion

**Figure 7:**
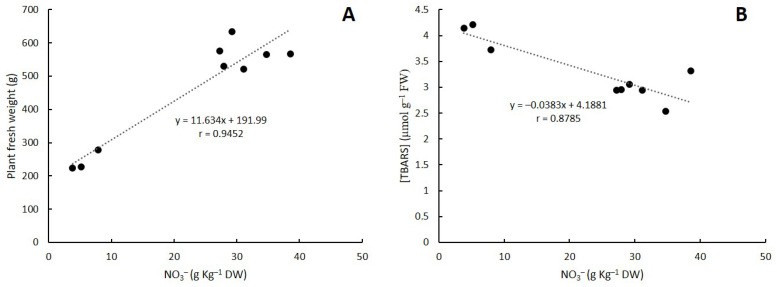
Relationship between plant fresh weight and NO_3_^−^ concentration (**A**) and between lipid peroxidation and NO_3_^−^ concentration (**B**) in kohlrabi plants irrigated with control solution (100S), 50% fish water + 50% drainage water (50F/50D) and 50% fish water + 50% drainage water + synthetic fertilizer (50F/50D + S); and inoculated with two formulations of plant growth-promoting rhizobacteria (Control, T1 and T2). Data are presented as treatment means (*n* = 6).

To date, this is the first study on the effect of PGPR, combined with an aquaponics system, on plant growth. Our results showed that the kohlrabi plants irrigated with the 50F/50D treatment had a reduced plant fresh weight as compared to the control. This reduction is an indicator that the restricted NO_3_^−^ supply in the 50F/50D treatment caused stress in these plants. In addition, a positive linear correlation was observed between the leaf content of NO_3_^−^ and fresh weight, which represents the contribution of NO_3_^−^ to the growth of kohlrabi plants in this study (r = 0.9452) (Figure 7). It is known that the availability of nutrients, mainly N, has significant effects on the agronomic characteristics, crop yield, and photosynthetic capacity of the leaves of various plants [14]. When the plants were irrigated with the 50F/50D + S treatment, this previously mentioned negative effect disappeared, and the concentration of NO_3_^−^ in these plants was even higher than in the control plants, while the weights were similar to those of the control plants. These data are extremely interesting, as this treatment maintained the levels of production at the same time that the consumption of water and fertilizers was reduced. 

Despite the strong reduction observed in the fresh weight of the plants irrigated with the 50F/50D treatment, the photosynthetic rate was not affected by the different N treatments. A similar result was observed by Cechin and Valquilha [14] in *Amaranthus* plants with a low N availability, which saw their shoot dry matter reduced by 74%, while photosynthesis was only reduced by 29%. This could be explained by the dependence of the photosynthetic capacity of plants on the photosynthetic rate and the photosynthetic area. Since the plants irrigated with the 50F/50D treatment were smaller (less photosynthetic area), CO_2_ assimilation was reduced, despite the maintenance of the photosynthetic rate. These data coincide with those observed by other researchers [15,16], who did not observe a significant correlation between crop yield and photosynthetic rate. Furthermore, Lawlor [17] revealed that leaf area development is much more sensitive to changing environmental conditions than photosynthesis.

In the literature, conflicting data have been found on the effect of organic fertilizers on photosynthetic parameters. Authors such as Efthimiadou et al. [18] reported increases in the photosynthetic rate when organic fertilizers were applied; however, the opposite or a null effect was observed by other authors such as Zhang et al. [19] and Liu et al. [20]. In our case, the 50F/50D + S treatment did not show significant differences, although there seemed to be a slight tendency for photosynthesis to increase in these plants as compared to the control plants. Therefore, we may think that the slight increase in the concentration of NO_3_^−^ in the 50F/50D + S treatment, which was slightly lower than the concentration in the control solution, favored photosynthesis. These data should be studied in future experiments.

In addition, as expected, the lower NO_3_^−^ supply to the plants led to a lower concentration of photosynthetic pigments (chlorophylls) in the kohlrabi leaves, which coincides with what was observed in other studies [14]. In general, the irrigation treatments had a greater impact on chl *b* than on chl *a*. Our results are also supported by Zhang et al. [19], who reported a lower leaf chlorophyll content in *Actinidia chinesis* when treatments with organic and inorganic fertilizers were tested, with N being the limiting nutrient instead of P or K, as indicated by Jiang et al. [21] and Zhao et al. [22]; however, in our case, the observed reduction in chl *b* again seemed to have been so slight that it had no effect on the photosynthetic rate.

It has been observed that some bioactive compounds (phenols and vitamin C) and soluble sugars tend to increase with a lower application of N [1]. These findings support our results, as the 50F/50D treatment, which contributed the lowest N concentration, resulted in kohlrabi plants with the highest foliar glucose, fructose, and sucrose content. This increase was due to the fact that soluble sugars participate in different biological processes and are important components in abiotic stress signaling, increasing the plant’s resistance [5]. Therefore, we suggest that the response of some sugars under N-deficient conditions in kohlrabi leaves is similar to that observed under other abiotic stresses.

However, interestingly in the case of total phenols, the reduction was only observed in the plants irrigated with the 50F/50D + S treatment, which barely showed differences in the concentration of NO_3_^−^ supplied with respect to the control. Conversely, lipid peroxidation was found to be negatively correlated with leaf NO_3_^−^ concentration (r = 0.8785) (Figure 7). This increase in lipid peroxidation, due to a lack of NO_3_^−^, was due to oxidative damage that appeared as a response to abiotic stress, which is consistent with what was observed by our group in another experiment carried out on cauliflower [23]. The stress that occurs in plants after exposure to NO_3_^−^-deficient nutrition induces the generation of reactive oxygen species, which can damage cell membranes, leading to lipid peroxidation. Therefore, plants have developed different defense mechanisms such as synthesis of protective enzymes (ascorbate peroxidase, superoxide dismutase, catalase and peroxidase) and antioxidant compounds (xanthophylls, carotenoids, polyphenols) in their cell membrane systems [23].

On the other hand, when we studied the NO_3_^−^ deficiency treatment combined with PGPR, we observed that T1 obtained the best results. Numerous studies with PGPR have observed a stimulating effect on the weight of the plant, and especially on the weight of the root, which could be attributed to the production of growth-promoting substances (ABA, IAA and various gibberellins) [24]. Authors such as Eduardo et al. [25] postulated that plants inoculated with *Azospirillum brasilense* had greater resistance to light, water and/or nutrient deficiencies among other abiotic stresses, which coincides with our results when applying a nitrogen deficiency to kohlrabi plants. It has been observed that this root growth could be responsible for the greater acquisition of both water and nutrients. These findings support our results, as we observed that the kohlrabi plants irrigated with the 50F/50D treatment and inoculated with T1 had increased concentrations of NO_3_^−^ and SO_4_^2−^ in the leaves. Since N plays a crucial role in photosynthetic efficiency [26], this increase in NO_3_^−^ concentration in the leaves of plants inoculated with T1 led to a higher photosynthetic rate of these plants, and consequently, a greater fresh weight of plants. Therefore, we can say that the application of T1 attenuated the negative effects caused by the 50F/50D treatment. In addition, the reduction in the glucose and fructose content, and the lipid peroxidation in plants irrigated with the 50F/50D treatment and inoculated with T1, corroborate the effect of this formulation in the attenuation of stress due to N deficiencies. These results are very interesting, because achieving a balance between the reduction in N input and the effect of the application of PGPB could translate into a reduction in the economic cost of the crop.

On the contrary, the inoculation with T2 did not generate any notable effects on the N-deficient plants. This could suggest that in the case of kohlrabi, it is necessary to select the genotype of the plant, because, as also observed by Lara et al. [27], in the case of sugarcane, the plant–bacteria interaction is associative, that is, there is a specific bacteria–cultivar interaction [28]. Something similar was observed by Sun et al. [29], who observed different responsiveness when inoculating two different varieties of corn with *Azospirillum*. In this case, there was a greater interaction between *Azospirillum brasilense* strain M3 and *Pantoea disperse* strain C3 with kohlrabi plants, than between *Azotobacter salinestris* and kohlrabi plants. Other authors such as Ahemad and Kibret [9] have associated the lack of effect of the PGPR with variable environmental conditions, such as climatic variations. Traits that promote plant growth do not work independently of each other, but rather additively or synergistically. Multiple mechanisms, such as phosphate solubilization, dinitrogen fixation, l-aminocyclopropane-l-carboxylate deaminase and antifungal activity, indole acetic acid and siderophore biosynthesis, etc. are responsible for the promotion of plant growth and increased yield [10].

## 4. Materials and Methods

### 4.1. Plant Material and Growth Conditions

Purple kohlrabi (*Brassica oleracea* var. *gongylodes*), cv. Ukza seedlings were obtained from a commercial nursery (Baby plant, S.L., Santomera, Spain), when the plants were 8–10 cm in height. They were grown in 1.2 m long bags (38.4 m^3^) filled with coconut fiber (Pelemix, Alhama de Murcia, Murcia, Spain). Each bag contained 3 plants and 3 drippers (2 L h^−1^). Excess nutrient solution was applied to produce a minimum of 30% drainage to avoid salt accumulation and nutrient imbalance in the rhizosphere [30]. Fourteen days after transplanting, 3 irrigation treatments were applied: (1) control solution (100S), which is a traditional solution used by local farmers and with 100% synthetic fertilizers; (2) 50% fish water/50% drainage water (reuse) (50F/50D); and (3) 50% fish water/50% drainage water + nutritional supplementation through the roots (50F/50D + S). The nutrient composition of the different solutions is shown in Table 1.

In addition, before transplanting, 15 g of *Azospirillum brasilense* strain M3 and *Pantoea dispersa* strain C3, immobilized in a solid support, with 10^9^ CFU g^−1^ (colony-forming units) (T1: Biopron^®^, Probelte SA., Murcia, Spain) was added to the substrate per each plant. The 15 g of T1 was applied to the first 15 cm of the substrate, right in the area where the plant was later placed. Thus, guaranteeing the proximity of the inoculum to the roots of the plant. In the same way, 10 mL of *Azotobacter salinestris* (at 250 µg mL^−1^), with 10^7^ CFU g^−1^ (T2: Nutribio N^®^, Ceres Biotics Tech, S.L., San Fernando de Henares, Madrid, Spain) was applied per plant, and the application was repeated every 2 weeks until the end of the experiment, following the manufacturer’s recommendation. This formulation was applied via a syringe to the substrate next to the stem of the plant (coinciding with the root zone). A total of 9 treatments were applied: 3 irrigation treatments (100S, 50F/50D, 50F/50D + S), and 3 PGPR treatments (control, T1 and T2) (see Table 2). Each treatment had a total of 27 plants, with a randomized distribution in 3 blocks inside the greenhouse. To carry out the analysis, 6 plants were harvested per treatment, taking 2 from each of the blocks.

**Scheme 1:**
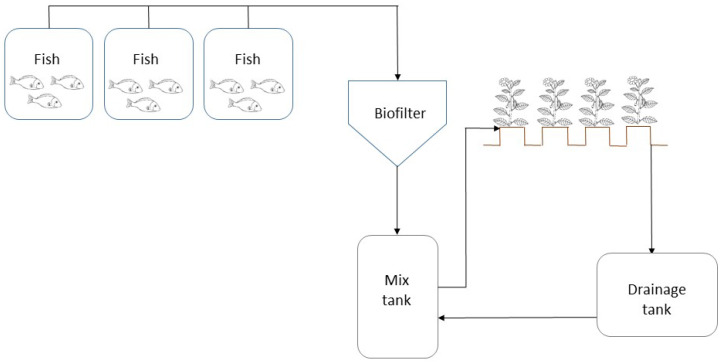
Schematic drawing of the aquaponics system.

This trial was carried out using a Recirculating Aquaponic System (RAS) installed in an existing greenhouse at the CIFEA facilities in Torre Pacheco (Murcia, Spain). The RAS produced fish for commercial purposes (*Oreochromis niloticus*), and the water was reused to irrigate the plants. The RAS was composed of 3 fish tanks of approximately 900 L each, a filter tank, a biofilter, a clarifier, and a collection tank where the drainage was collected (Scheme 1). The biological nitrification processes took place in the biofilter, due to the action of aerobic bacteria (genera *Nitrobacter* spp. and *Nitrosomonas* spp.), which oxidized the toxic products excreted by the fish (NH_3_) to NO_3_^−^.

A total of 213 fish distributed among the 3 tanks were used to carry out the experiment. The fish at the beginning of the experiment had an average weight of approximately 157 g, weighing 324 g at the end. For feeding, an amount of feed equivalent to 2.2–1.9% of the fish weight was provided daily (feed composition can be found in Supplementary Table S1).

Thirty-seven days after transplanting, kohlrabi plants were harvested and weighed to determine the fresh weight (PF) of the whole plant and of the leaves alone.

### 4.2. Ion Determination

The NO_3_^−^, SO_4_^−^, PO_4_^3−^ and Cl^−^ concentrations were measured from lyophilized and ground kohlrabi leaves (0.4 g) with 20 mL of deionized water. The mixture was shaken for 30 min. They were determined in an ion chromatograph (METROHM 861 Advanced Compact IC, Herisau, Switzerland; METROHM 838 Advanced Sampler, Herisau, Switzerland), and the column used was a METROHM Metrosep A Supp7 250/4.0 mm (METROHM, Herisau, Switzerland). The flow rate was 0.7 mL min^−1^, and the column temperature was kept at 45 °C. The solvent system consisted of Na_2_CO_3_ 3.6 mM, with an isocratic gradient.

### 4.3. Gas Exchange

The net CO_2_ assimilation rate (Pn), internal CO_2_ concentration (Ci), transpiration rate (E) and stomatal conductance (gs) were measured in the youngest fully open leaf of each plant, using a CIRAS-2 Portable Photosynthesis system (Amesbury, MA, USA) with a PLC6 (U) Automatic Universal Leaf Cuvette, measuring both sides of the leaves. The cuvette provided light (LED) with a photon flux density of 1300 m^−2^s^−1^, a [CO_2_] of 400 ppm, a leaf temperature of 28 °C, and 70% relative humidity. The water use efficiency (WUE) of the leaf gas exchange was calculated from the gas exchange data as Pn/E, where Pn is the carbon assimilated through photosynthesis, and E is the amount of water lost via transpiration.

### 4.4. Chlorophylls

To obtain chlorophylls, 0.5 g of frozen leaf was homogenized with an acetone-hexane solution (2:3) (25 mL). They were then centrifuged at 3500× *g* for 6 min at 4 °C, and the absorbance of the supernatant was measured with a spectrophotometer (Shimadzu UV-1800 model with the CPS-240 cell holder, Shimadzu Europa GmbH, Duisburg, Germany) at 663, 645, 505 and 453 nm. The concentration of chlorophyll *a* (Chl *a*), *b* (Chl *b*) and *a* + *b* (Chl *a* + *b*) were obtained using the formulas of Nagata and Yamashita [31].

### 4.5. Total Soluble Sugars

Soluble sugars were extracted according to Balibrea et al. [32], with slight modifications. Lyophilized kohlrabi leaf samples (50 mg) were incubated twice in 1.5 mL of methanol (80%, *v*/*v*), for 30 min each time, at 4 °C. During that time, the mixture was shaken thrice. Subsequently, each extract was centrifuged for 15 min at 3500× *g*, at 4 °C, and the supernatant was filtered through a C18 Sep-Pak cartridge (Waters Associates, Milford, MA, USA), previously activated with 20 mL of methanol/water (80/20). Then, the two supernatants from the double extraction were combined and filtered through a 0.45 μm filter (Millipore, Bedford, MA, USA). The concentration of glucose, fructose, and sucrose in the extracts were determined directly by ion chromatography, using an 817 Bioscan system (Metrohm, Herisau, Switzerland) equipped with a pulsed amperometric detector (PAD) and a gold electrode. The column used was a METROHM Metrosep Carb 1–150 IC column (METROHM, Herisau, Switzerland) (4.6 × 250 mm), which was heated to 32 °C.

### 4.6. Lipid Peroxidation and Total Phenolic Compounds

Lipid peroxidation was measured as the amount of thiobarbituric acid-reactive substances (TBARS), as determined by the thiobarbituric acid (TBA) reaction [33]. Lyophilized samples (0.1 g) were homogenized in 3 mL of 20% (*w*/*v*) trichloroacetic acid (TCA). The homogenate was centrifuged at 3500× *g* for 20 min. To a 1.5 mL aliquot of the supernatant, 1.5 mL of 20% (*w*/*v*) TCA containing 0.5% (*w*/*v*) TBA and 0.15 mL of 4% (*w*/*v*) butylated hydroxytoluene (BHT) in ethanol were added. The mixture was heated at 95 °C for 30 min and then quickly cooled on ice. Then, it was centrifuged at 10,000× *g* for 15 min, and the absorbance measured at 532 nm. The value for non-specific absorption at 600 nm was subtracted. The concentration of TBARS was calculated using an extinction coefficient of 155 mM^−1^ cm^−1^ [34].

The total phenolic compounds (TPC) were extracted from 0.5 g of the youngest full-sized leaves (frozen at −80 °C) with 5 mL of 80% acetone. The homogenate was centrifuged at 10,000× *g* at 4 °C, for 10 min. The Folin–Ciocalteu reagent was used, diluted with Milli-Q water (1:10): 1 mL of the diluted reagent was mixed with 100 μL of supernatant and 2 mL of Milli-Q water, to which 5 mL of sodium carbonate (20%) was then added. The mixture was kept for 30 min in the dark. The absorbance was measured at 765 nm, according to the methodology of Kahkonen et al. [35]. The total phenolic content was expressed as gallic acid equivalents, in mg g^−1^ dry weight. Analyses were carried out in 4 replicates.

### 4.7. Statistical Analysis

Data were tested for homogeneity of variance and normality of distribution. The significance of the treatment effects was determined using the SPSS 13.0 software package (IBM SPSS Statistics 25.0, Armonk, NY, USA), with an ANOVA Duncan’s multiple range test (*p* ≤ 0.05), using the treatments as a statistical variable to determine significant differences between means. For the quantification of different parameters, 6 samples per treatment were analyzed. Pearson’s correlation coefficient was used to explore the statistical relationship between the NO_3_^−^ concentration and plant fresh weight and lipid peroxidation.

## 5. Conclusions

The results discussed in this work clearly indicate that combining T1 and irrigation with organic (fish water) and inorganic (drainage water) fertilizers attenuated the negative effects caused by the N deficiency. These plants presented a higher foliar NO_3_^−^ concentration, which was shown as a higher photosynthetic rate, less sugar accumulation, less lipid peroxidation, and therefore, greater growth; however, we consider it necessary to continue studying different proportions of the mixture of fish water and drainage water, to not cause such a severe reduction in growth. On the contrary, T2, at least in this experiment, did not have significant effects on kohlrabi plants.

Another interesting finding was the 50F/50D + S treatment, which hardly showed any differences with the 100S treatment. This could indicate that this type of farming system (aquaponics) is viable for producing kohlrabi plants with a lower consumption of fertilizers and water, without reducing production.

## Figures and Tables

**Table 1:** Nutritional composition of macronutrients of the different solutions used for kohlrabi plants growth.

Nutrient (mg L^−1^)	Control	50F/50D	50F/50D + S
NO_3_^−^	606.70 ± 54.19	249.74 ± 38.27	550.22 ± 36.31
PO_4_^3−^	367.73 ± 10.50	290.54 ± 8.09	445.75 ± 7.97
SO_4_^2−^	380.46 ± 4.24	338.76 ± 2.19	392.04 ± 4.48
K^+^	458.44 ± 12.32	193.32 ± 7.59	429.89 ± 7.15
Ca^2+^	76.70 ± 3.07	80.61 ± 2.86	81.06 ± 2.84
Mg^2+^	65.68 ± 0.97	66.20 ± 0.96	68.60 ± 1.29

**Table 2:** Irrigation and PGPR treatment applied to kohlrabi plants.

Irrigation	PGPR	Treatments
100S	0	100S
	T1	100S + T1
	T2	100S + T2
50F/50D	0	50F/50D
	T1	50F/50D + T1
	T2	50F/50D + T2
50F/50D + S	0	50F/50D + S
	T1	50F/50D + S + T1
	T2	50F/50D + S + T2

## Data Availability

Data is contained within the article and supplementary materials.

## Supplementary Materials

The following are available online at https://www.mdpi.com/article/10.3390/plants13050595/s1, Table S1: The fish feed composition.
