# Supplementary material for: Plant Growth-Promoting Rhizobacteria as Tools to Improve the Growth of Kohlrabi (Brassica oleracea var. gongylodes) Plants in an Aquaponics System
Source: Plants (Basel). 2024 Feb 22;13(5):595. doi: 10.3390/plants13050595 (PMC10934560; doi:10.3390/plants13050595)
Supplement: Supplementary file 1 [file plants-13-00595-s001.zip › plants-2731200-supplementary.pdf]

Supplementary

Supplementary Table S1. The fish feed composition.

**Composition**	
Crude protein	32%
Crude fat	6%
Crude fibre	3.9%
Ash	6%
Calcium	1%
Sodium	0.2%
Phosphorus	0.8%
**Additives**	
E672 Vitamin A	5000 U.I/I.E.
E671 Vitamin D3	750 U.I/I.E.
E1 Iron	42 mg
E2 Iodine	2.1 mg
E4 Copper	5 mg
E5 Manganese	16 mg
E6 Zinc	100 mg
E321 BHT	50 mg
